# The Potential Protective Effect of Oligoribonucleotides-d-Mannitol Complexes against Thioacetamide-Induced Hepatotoxicity in Mice

**DOI:** 10.3390/ph11030077

**Published:** 2018-08-06

**Authors:** Tetiana Marchyshak, Tetiana Yakovenko, Igor Shmarakov, Zenoviy Tkachuk

**Affiliations:** 1 Institute of Molecular Biology and Genetics, National Academy of Sciences of Ukraine, 03680 Kyiv, Ukraine; biochem.imbg@gmail.com (T.M.); tetianayakovenko46@gmail.com (T.Y.); 2 Department of Biochemistry and Biotechnology, Yurii Fedkovych Chernivtsi National University, 58012 Chernivtsi, Ukraine; igor.shmarakov@gmail.com

**Keywords:** oligoribonucleotides-d-mannitol complexes, thioacetamide-induced hepatotoxicity, hepatoprotective effect

## Abstract

This study investigated the potential hepatoprotective effect of oligoribonucleotides-d-mannitol complexes (ORNs-d-M) against thioacetamide (TAA)-induced hepatotoxicity in mice. The hepatoprotective activity of ORNs-d-M was evaluated in thioacetamide (TAA)-treated C57BL/6J. Results indicate that treatment with ORNs-d-M displayed a protective effect at the TAA-induced liver injury. Treatment with ORNs-d-M, starting at 0 h after the administration of TAA, decreased TAA-elevated serum alanine aminotransferase (ALT) and γ-glutamyl transpeptidase (GGT). Activities of glutathione S-transferase (GST) and glutathione peroxidase (GPx), and levels of glutathione (GSH), were enhanced with ORNs-d-M administration, while the hepatic oxidative biomarkers (TBA-reactive substances, protein carbonyl derivatives, protein-SH group) and myeloperoxidase (MPO) activity were reduced. Furthermore, genetic analysis has shown that the ORNs-d-M decreases the expression of mRNA pro-inflammatory cytokines, such as tumor necrosis factor α (TNF-α) and interleukin-6 (IL-6), profibrogenic cytokine-transforming growth factor β1 (TGF-β1), as well as the principal protein of the extracellular matrix—collagen I. The present study demonstrates that ORNs-d-M exerts a protective effect against TAA-induced liver injury, which may be associated with its anti-inflammatory effects, inhibition of overexpression of mRNA cytokines, and direct effects on the metabolism of the toxin.

## 1. Introduction

The liver acts as the main metabolic organ where the major biochemical processes involved in maintaining the homeostasis of an organism are integrated. Being a detoxifying organ, the liver is the first to make contact with xenobiotics, the action of which causes serious disorders, which lead to the development of the pathological process [1]. Toxins and drugs are among the main etiopathogenetic agents causing an acute hepatic failure. Acute toxic liver injury is characterized by membrane damage, oxidative stress, massive necrosis of hepatocytes, infiltration of parenchyma by neutrophils and activation of hepatic stellate cells, following an increased inflammation and damage to the liver [2]. This is a key event in inducing liver damage. It is generally accepted that the molecules involved in the initiation of hepatic inflammation include pro-inflammatory mediators TNF-α, cyclooxygenase-2 and IL-6, which trigger the cascade of cytokines mediating the inflammatory reactions [3]. One of the key events of acute hepatotoxicity is an activation of hepatic stellate cells (HSC). Activated stellate cells (of myofibroblast type) are characterized by a high level of proliferation, migration and contractility [4]. These activated cells are capable of expressing the major profibrotic factor-transforming growth factor β1. In addition, activated hepatic stellate cells overexpress the components of the extracellular matrix (ECM), including fibril-forming type I and III collagens, and laminin. This overexpression leads to the deposition of collagen contributing to the development of liver fibrosis [5].

Antisense RNAs, aptamers and modified nucleic acids have been used in the treatment of liver disease, which acts by binding to target nucleic acids or proteins [6]. Using antisense nucleotides targeted towards mRNA encoding TNF-α and TGF-β1 has been shown to prevent hepatic damage effectively against the action of toxic substances [7,8].

Natural oligoribonucleotides (ORNs) (total yeast RNA) have long attracted attention as pharmacological agents. They possess a wide spectrum of biological activity. It is known that the ORNs have anti-inflammatory effects investigated on local inflammation models and membrane-stabilizing action [9,10]. It was shown in a study that the oligoribonucleotides-d-mannitol complexes (ORNs-d-M) possess antiviral activity against the hepatitis C virus. Application of complexes in the treatment of hepatitis C reduces the concentration of the virus in the bloodstream and normalizes phagocytic index of monocytes [11,12,13]. Since hepatitis C leads to acute and chronic hepatitis, fibrosis, cirrhosis and hepatocellular carcinomas, the search for drugs with antiviral and hepatoprotective activities is promising. That is why the aim of this study was to investigate the hepatoprotective effect of the ORNs-d-M complexes at thioacetamide-induced acute hepatotoxicity.

In this study, it was discovered that the ORNs-d-M have hepatoprotective effects, which are manifested in reducing the oxidative stress and inflammation.

## 2. Results

### 2.1. Influence of the ORNs-d-M on Indicators of Inflammation and Liver Damage at Acute Hepatotoxicity

**Figure 1:**
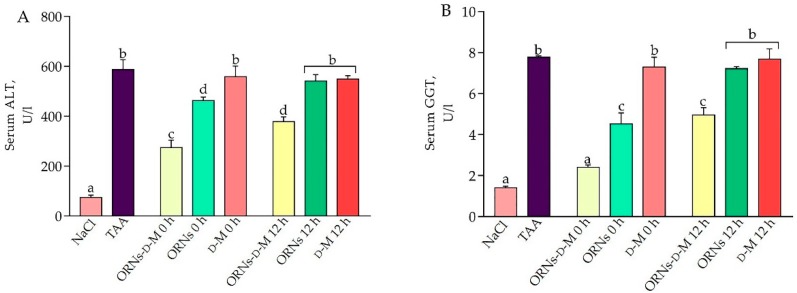
Effects of oligoribonucleotides-d-mannitol complexes (ORNs-d-M), natural oligoribonucleotides (ORNs) and d-mannitol (D-M) on the activities of serum ALT (**A**) and GGT (**B**) in TAA-induced liver damage mice. Data are presented as the mean ± standard deviation (SD), *n* = 6 for each group. Values marked with different letters (a, b, c, d) are statistically different, *p* < 0.05 (Student’s *t*-test). Abbreviations: NaCl—control mice receiving a single injection of NaCl; TAA—mice receiving a single injection of TAA; ORNs-d-M 0 h—mice receiving a TAA and ORNs-d-M immediately after TAA application (i.e., 0 h) and every next 12 h for 48 h; ORNs 0 h—mice receiving a TAA and ORNs immediately after TAA application (i.e., 0 h) and every next 12 h for 48 h; D-M 0 h—mice receiving a TAA and then D-M immediately after TAA application (i.e., 0 h) and every next 12 h for 48 h; ORNs-d-M 12 h—mice receiving a TAA and ORNs-d-M after 12 h after TAA application (i.e., 12 h) and every next 12 h for 48 h; ORNs 12 h—mice receiving a TAA and ORNs after 12 h after TAA application (i.e., 12 h) and every next 12 h for 48 h; D-M 12 h—mice receiving a TAA and D-M after 12 h after TAA application (i.e., 12 h) and every next 12 h for 48 h.

It is well known that alanine aminotransferase (ALT) and γ-glutamyl transpeptidase (GGT) are sensitive biochemical markers in the serum for acute liver damage. Results of the liver function detection are shown in Figure 1A,B. The thioacetamide (TAA)-treated group demonstrated a notable elevation of ALT and GGT levels in serum compared to the control group (*p* < 0.05). By contrast, ALT levels reduced by 53.3% and 35.6%, respectively, in the groups of animals receiving the first dose of ORNs-d-M at 0 h (ORNs-d-M 0 h) and at 12 h after the administration of toxin (ORNs-d-M 12 h). Also, treatment with ORNs-d-M contributed to a significant reduction in serum GGT levels in both experimental groups. It was also found out that the co-administering of ORNs with TAA (ORNs 0 h) reduces ALT and GGT levels. Conversely, at treatment with ORNs 12 h after the start of the study (ORNs 12 h group) and d-mannitol (d-M 0 h and d-M 12 h groups), the levels of ALT and GGT did not significantly differ from the TAA-treated group. Thus, we assume that the protective effect of ORNs-d-M in the model of TAA-induced acute liver damage is due to their effect on the metabolism of the toxin.

**Figure 2:**
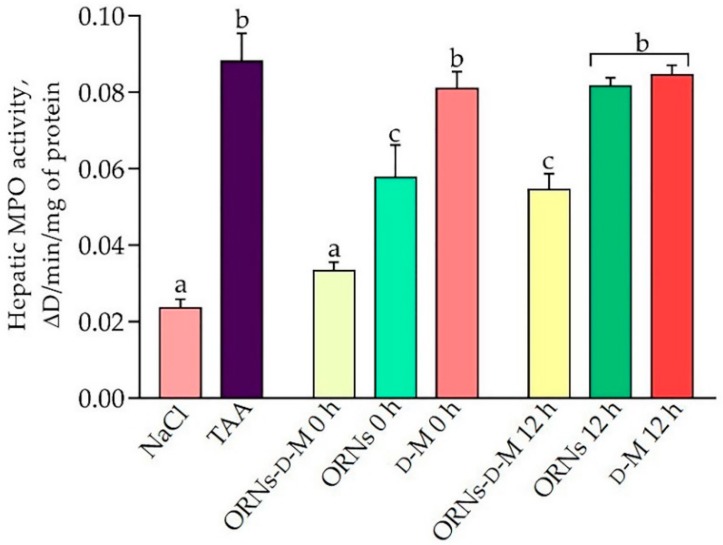
Effects of ORNs-d-M, ORNs and d-M on the MPO activity in TAA-induced liver damage mice. Data are presented as the mean ± SD, *n* = 6 for each group. Values marked with different letters (a, b, c, d) are statistically different, *p* < 0.05 (Student’s *t*-test). Abbreviations: NaCl—control mice receiving a single injection of NaCl; TAA—mice receiving a single injection of TAA; ORNs-d-M 0 h—mice receiving a TAA and ORNs-d-M immediately after TAA application (i.e., 0 h) and every next 12 h for 48 h; ORNs 0 h—mice receiving a TAA and ORNs immediately after TAA application (i.e., 0 h) and every next 12 h for 48 h; d-M 0 h—mice receiving a TAA and then d-M immediately after TAA application (i.e., 0 h) and every next 12 h for 48 h; ORNs-d-M 12 h—mice receiving a TAA and ORNs-d-M after 12 h after TAA application (i.e., 12 h) and every next 12 h for 48 h; ORNs 12 h—mice receiving a TAA and ORNs after 12 h after TAA application (i.e., 12 h) and every next 12 h for 48 h; d-M 12 h—mice receiving a TAA and d-M after 12 h after TAA application (i.e., 12 h) and every next 12 h for 48 h.

To confirm the anti-inflammatory effects of the complexes, the myeloperoxidase (MPO) activity was investigated as an indicator of inflammatory infiltration of liver parenchyma by neutrophils [14]. The MPO activity results, after ORNs-d-M, ORNs and D-M administration into the mice of various treatment groups, are shown in Figure 2. The administration of TAA significantly increased MPO activity compared to the control group of animals. At the same time, ORNs-d-M 0 h, ORNs-d-M 12 h and ORNs 0 h groups showed a significant reduction in MPO activity by 58%, 39% and 35%, respectively, compared to the group of animals receiving TAA. Conversely, MPO activity in groups of ORNs 12 h, D-M 0 h and D-M 12 h decreased insignificantly in comparison with the TAA-treated group.

### 2.2. Influence of the ORNs-d-M on Oxidative Stress in the Liver

**Figure 3:**
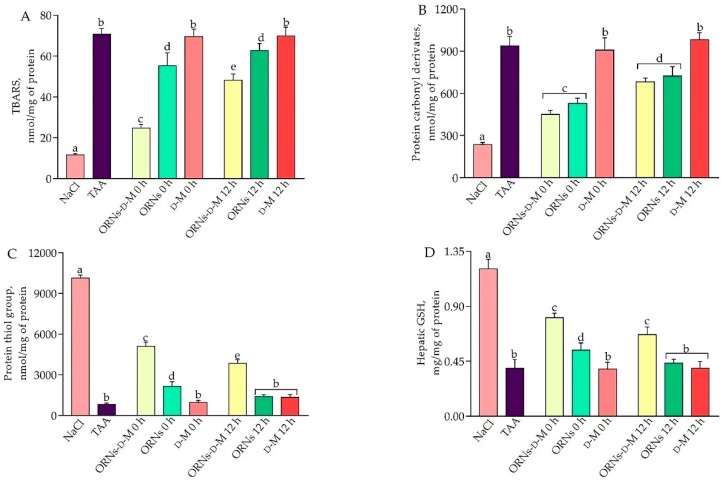
Effects of ORNs-d-M, ORNs and D-M on the levels of thiobarbituric acid reactive substances (TBARS) (**A**), protein carbonyl derivatives (**B**), protein thiol groups (**C**) and glutathione (GSH) (**D**) in TAA-induced liver damage mice. Data are presented as the mean ± SD, *n* = 6 for each group. Values marked with different letters (a, b, c, d, e) are statistically different, *p* < 0.05 (Student’s *t*-test). Abbreviations: NaCl—control mice receiving a single injection of NaCl; TAA—mice receiving a single injection of TAA; ORNs-d-M 0 h—mice receiving a TAA and ORNs-d-M immediately after TAA application (i.e., 0 h) and every next 12 h for 48 h; ORNs 0 h—mice receiving a TAA and ORNs immediately after TAA application (i.e., 0 h) and every next 12 h for 48 h; d-M 0 h—mice receiving a TAA and then d-M immediately after TAA application (i.e., 0 h) and every next 12 h for 48 h; ORNs-d-M 12 h—mice receiving a TAA and ORNs-d-M after 12 h after TAA application (i.e., 12 h) and every next 12 h for 48 h; ORNs 12 h—mice receiving a TAA and ORNs after 12 h after TAA application (i.e., 12 h) and every next 12 h for 48 h; d-M 12 h—mice receiving a TAA and d-M after 12 h after TAA application (i.e., 12 h) and every next 12 h for 48 h.

Hepatotoxicity TAA requires metabolic activation with the formation of reactive metabolites S-oxide (TASO) and S,S-dioxide (TASO_2_) [15]. Because increased levels of reactive oxygen species (ROS) induce lipid and protein oxidative damage, the levels of products of lipid peroxidation, protein carbonyl derivatives, protein thiol groups and reduced glutathione were investigated. The results of the research are shown in Figure 3. As shown in Figure 3A,B, the acute toxic liver injury was characterized by an increase in the level of TBA-reactive substances (TBARS) and protein carbonyl derivatives by 6.1 and 3.97 times, respectively, compared with the control group. At the same time, the levels of protein thiol groups and reduced glutathione (GSH) in TAA-induced mice livers were found to be 91.9% and 67.5% lower than the control group (Figure 3C,D). However, the treatment with ORNs-d-M 0 h, ORNs-d-M 12 h, ORNs 0 h and ORNs 12 h significantly (*p* < 0.05) reduced TBARS and protein carbonyl derivatives compared to the TAA group (Figure 3A,B). The levels of protein thiol groups and GSH significantly increased in ORNs-d-M 0 h and ORNs-d-M 12 h groups in comparison with the TAA-treated group (Figure 3C,D). In turn, ORNs 0 h and ORNs 12 h did not have a significant effect on the levels of protein thiol groups and reduced glutathione.

### 2.3. Influence of the ORNs-d-M on GST and GPx Activities at Acute Toxic Liver Injury

After 48 h of intoxication of mice with TAA and treatment with ORNs-d-M, ORNs and d-M, the activities of glutathione S-transferase (GST) and glutathione peroxidase (GPx) were investigated. As shown in Table 1, the intoxication of mice with TAA significantly decreased the activities of oxidative stress marker enzymes in the liver, including GST and GPx, by 43.8% and 46.9%, respectively, in comparison with control group. However, the downregulated activities of GST and GPx after TAA administration were significantly elevated by ORNs-d-M 0 h, ORNs-d-M 12 h and ORNs 0 h. Conversely, the activities of GST and GPx remained unchanged in groups of the treated mice with ORNs 12 h, d-M 0 h and d-M 0 h in comparison with the control. 

### 2.4. Influence of the ORNs-d-M on Expression of IL-6, TNF-α, TGF-Β1, COL1A1 and α-SMA mRNA at Acute Hepatotoxicity

**Figure 4:**
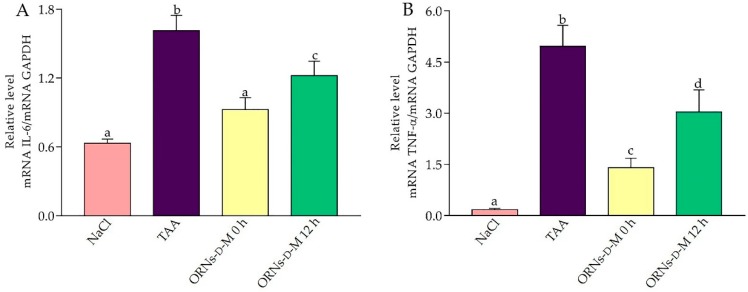
Effects of ORNs-d-M on the expression of IL-6 (**A**) and TNF-α (**B**) mRNA in TAA-induced liver damage mice. The investigated mRNA levels were normalized to GAPDH as a control. Data are presented as the mean ± SD, *n* = 6 for each group. Values marked with different letters (a, b, c, d) are statistically different, *p* < 0.05 (Student’s *t*-test). Abbreviations: NaCl—control mice receiving a single injection of NaCl; TAA—mice receiving a single injection of TAA; ORNs-d-M 0 h—mice receiving a TAA and ORNs-d-M immediately after TAA application (i.e., 0 h) and every next 12 h for 48 h; ORNs-d-M 12 h—mice receiving a TAA and ORNs-d-M after 12 h after TAA application (i.e., 12 h) and every next 12 h for 48 h.

It is known that oxidative stress stimulates the pro-inflammatory reactions in liver parenchyma, and therefore, the expression of IL-6 and TNF-α genes expressed by resident macrophages of the liver and recruited cells of the immune system [16] has been investigated. Single administration of TAA was detected to increase the mRNA level of the pro-inflammatory cytokines IL-6 and TNF (Figure 4A,B). However, ORNs-d-M suppressed the increase of these cytokines induced by the acute toxic dose of TAA, depending on the time of treatment with complexes. The TNF-α and IL-6 levels were significantly different between ORNs-d-M 0 h, ORNs-d-M 12 h and TAA groups (*p* < 0.05).

**Figure 5:**
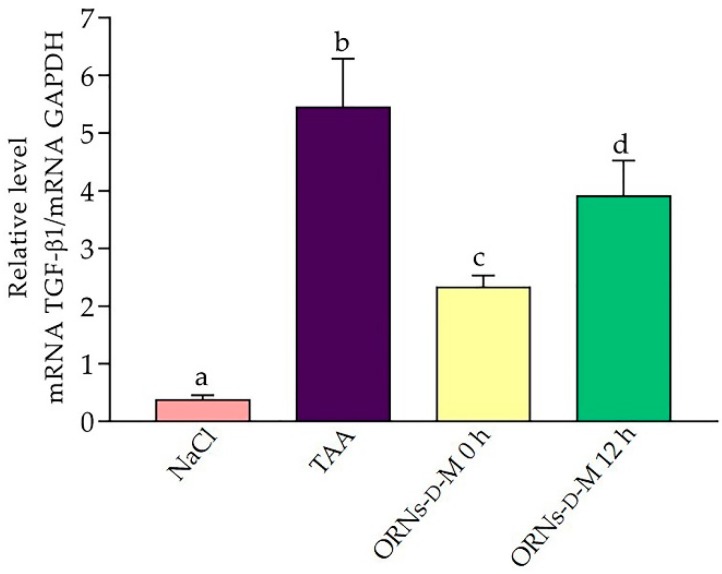
Effect of ORNs-d-M on the expression of TGF-β1 mRNA in TAA-induced liver damage mice. The investigated mRNA levels were normalized to GAPDH as a control. Data are presented as the mean ± SD, *n* = 6 for each group. Values marked with different letters (a, b, c, d) are statistically different, *p* < 0.05 (Student’s *t*-test). Abbreviations: NaCl—control mice receiving a single injection of NaCl; TAA—mice receiving a single injection of TAA; ORNs-d-M 0 h—mice receiving a TAA and ORNs-d-M immediately after TAA application (i.e., 0 h) and every next 12 h for 48 h; ORNs-d-M 12 h—mice receiving a TAA and ORNs-d-M after 12 h after TAA application (i.e., 12 h) and every next 12 h for 48 h.

The activation of stellate cells of liver is defined as the central event in the development of liver fibrosis. TGF-β1 acts as one of the major profibrotic cytokines in liver disease [17]. We detected an increase in the expression of this gene by 15.1 times in TAA-injured livers compared with the control group (Figure 5). However, in the ORNs-d-M 0 h and ORNs-d-M 12 h groups, the TGF-β1 mRNA expression level was significantly decreased by 57% and 28.4%, respectively, in comparison with the TAA-treated group.

**Figure 6:**
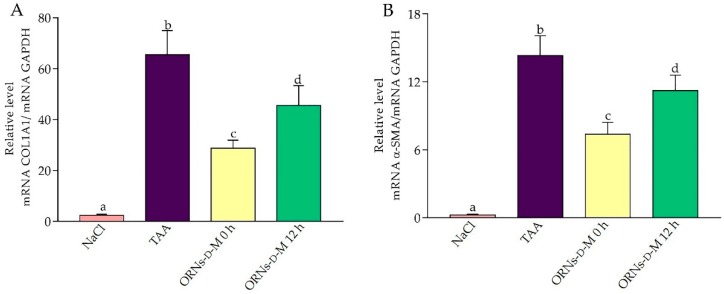
Effects of ORNs-d-M on the expression of COL1A1 (**A**) and α-SMA (**B**) mRNA in TAA-induced liver damage mice. The investigated mRNA levels were normalized to GAPDH as a control. Data are presented as the mean ± SD, *n* = 6 for each group. Values marked with different letters (a, b, c, d) are statistically different, *p* < 0.05 (Student’s *t*-test). Abbreviations: NaCl—control mice receiving a single injection of NaCl; TAA—mice receiving a single injection of TAA; ORNs-d-M 0 h—mice receiving a TAA and ORNs-d-M immediately after TAA application (i.e., 0 h) and every next 12 h for 48 h; ORNs-d-M 12 h—mice receiving a TAA and ORNs-d-M after 12 h after TAA application (i.e., 12 h) and every next 12 h for 48 h.

Analysis of hepatic collagen mRNA expression revealed a strong induction of collagen after 48 h in the animal group, which received a single TAA injection. Nevertheless, in ORNs-d-M 0 h and ORNs-d-M 12 h groups, this figure was significantly (*p* < 0.05) lower than in the TAA-treated group (Figure 6A). Additionally, the expression of the α-SMA gene, which is expressed by activated stellate cells, has been investigated. Toxin administration increased the expression mRNA level of α-SMA by 59.7 times compared with the control group (Figure 6B). In contrast, the ORNs-d-M 0 h and ORNs-d-M 12 h reduced the expression mRNA level of α-SMA after TAA application, depending on the time of treatment with complexes.

## 3. Discussion

The liver is the main metabolic and detoxifying organ that first contacts and neutralizes xenobiotics [18]. Despite the presence in the hepatocytes of the cellular system of detoxification (cytochrome P450, flavin-containing monooxygenase, glutathione transferase, glucuronyltransferase), which provide biotransformation of xenobiotics, the metabolic transformations of some of them can lead to the formation of toxic intermediates, resulting in a liver toxic injury [19]. TAA is an organosulfur compound, which today is used as a fungicidal agent and organic solvent in the leather, textile and paper industries [20]. At the same time, it acts as hepatotoxin for the induction of acute hepatic failure. The mechanism by which TAA causes the liver injury involves its biotransformation using the cytochrome P450 enzyme system. These enzymes convert TAA to thioacetamide sulfoxide (TAASO), a reactive intermediate with toxic nature, and then to a more reactive thioacetamide-S,S-dioxide (TAASO_2_), causing severe damage to the liver [21,22].

Liver injury can be assessed by monitoring sensitive biochemical markers such as ALT and GGT, which are released from the hepatocytes into the circulatory system by changing membrane permeability. In this study, TAA leads to increased GGT and ALT activities in blood serum of mice, indicating a loss of functional integrity of hepatocytes [23]. When ORNs-d-M was applied, the levels of these marker enzymes were restored, depending on the start time of the complexes’ administration (at 0 h or 12 h after hepatotoxin application). ORNs-d-M treatment from the beginning of the study (ORNs-d-M 0 h) showed a profound hepatoprotective effect that was not observed in ORNs 12 h and d-mannitol groups (D-M 0 h and D-M 12 h). These data allow us to argue that ORNs-d-M, but not ORNs, have a protective effect in the model of TAA-induced liver damage. Restoring lesions may be due to hepatocyte repair and stabilization of the cell membrane by the ORNs-d-M. Previously, it had been shown that ORNs-d-M have membrane-stabilizing properties in the in-vitro system [10]. Therefore, we assume that ORNs-d-M can stabilize the structural integrity of the hepatocellular membrane at TAA-induced liver damage.

TAA treatment promotes necrosis and apoptosis of hepatocytes [22], which in turn is accompanied by the recruitment of neutrophils to liver parenchyma. One of the major molecules released after the recruitment of neutrophils is myeloperoxidase, an important enzyme involved in the generation of active forms of oxygen, and it may further damage the parenchyma [24]. It has been found that treatment with ORNs-d-M, starting at 0 and 12 h after the administration of hepatotoxin, significantly reduced MPO activity in liver parenchyma, but not ORNs and D-M. The obtained results suggest that ORNs-d-M can reduce inflammatory infiltration of parenchyma by neutrophils.

The active intermediates TAA (TAASO, TAASO_2_) formed by the pro-oxidant free-radical mechanism lead to the formation of adducts of proteins, lipids and nucleic acids. The formation of reactive oxygen species (ROS), which is the result of the TAA activation, leads to the initiation of processes of lipid and protein peroxidation, damage to mitochondria and violation of energy generation [25,26]. We evaluated the ability of the ORNs-d-M to affect the levels of oxidative stress markers in TAA-damaged livers, and detected that ORNs-d-M treatment from 0 h after TAA administration decreases the levels of oxidative stress markers in TAA-treated mice. Interestingly, the treatment with complexes starting from 12 h after TAA administration showed a weaker protective effect compared with the group receiving complexes from the beginning of the study (ORNs-d-M 0 h). It is known that the ORNs-d-M based on total yeast RNA possesses anti-inflammatory properties, inhibits oxidative processes in cell membranes and contributes to the normalization of inducible nitric oxide synthase activity [9,10,27]. Therefore, on the one hand, we assume that the resulting effect may be related to the above-mentioned ORNs-d-M properties. On the other hand, the resulting effect in the ORNs-d-M 12 h group leads us to believe that complexes can directly affect the metabolism of TAA, thus preventing the development of hepatotoxicity.

One of the main mechanisms of TAA-induced liver injury is the formation of free radicals in TAA metabolism [20]. Consequently, antioxidant activity and inhibition of free-radical production are important for protecting cells from TAA-induced hepatotoxicity. GSH is a known antioxidant, which by binding covalently to free radicals and enhancing the activity of glutathione peroxidase and glutathione reductase plays an important role against TAA-induced acute liver damage [28,29]. Our results have shown that TAA overdose results in the depletion of a GSH pool. However, treatment with ORNs-d-M, starting at 0 and 12 h after the administration of hepatotoxin, significantly increased GSH levels compared to the TAA-treated group. Antioxidant enzymes such as GPx and GST perform important functions in protection mechanisms against the harmful effects of free radicals in the liver. This study showed that the ORNs-d-M protects the liver from free radical oxidation by enhancing the activity of the glutathione-related antioxidant defense system. The ORNs-d-M probably acted by increasing the level of reduced glutathione. Depletion of the glutathione pool by oxidative stress affects the activity of glutathione-dependent enzymes that neutralize ROS. The ORNs-d-M could restore depleted levels of GSH and thereby increase the GPx and GST activities. Thus, this study showed that ORNs-d-M effectively protects the liver by improving the enzymatic and nonenzymatic protection against acute toxic liver damage.

The series of factors that are involved in the liver injury pathogenesis include oxidative stress, inflammation and immune responses. Cytokines act as central mediators that link with specific receptors on the cell membrane of targeting cells, thereby causing a cascade of reactions leading to induction, amplification or inhibition of the activity of the genes regulated by them [30]. TNF-α and IL-6 are important hepatotoxic mediators when liver is damaged by the toxins. It has been shown that the use of TAA induces the production of the nuclear factor-kappa B (NF-κB), which leads to the production of various inflammatory factors, including TNF-α and IL-6 [31,32]. NF-κB activation would contribute to massive hepatocyte death, inflammation, and activation of hepatic stellate cells [33]. TNF-α acts as an important inflammatory mediator that is able to modulate the effects of other cytokines, such as IL-6 [34]. In this study, treatment with ORNs-d-M, starting with both 0 and 12 h after the administration of hepatotoxin, reduced the increased expression of both the TNF-α and IL-6 mRNA. Reduced expression of TNF-α mRNA by the action of complexes indicates a reduced activation of Kupffer cells and liver infiltration by immune system cells, which has been confirmed by biochemical parameters (MPO activity). Since NF-κB is a central transcription mediator, which regulates the expression of inflammatory cytokines (TNF-α, IL-6), we assume that complexes may suppress NF-kb activation, which leads to reducing TNF-α and IL-6 mRNA expression.

HSC activation is recognized as a central event in the development of hepatic fibrosis and occurs early in response to liver injury. Therefore, in the model of acute TAA-induced liver injury, the activation of stellate liver cells was also described as a critical event by us and others [35,36]. Activated hepatic stellate cells are capable of ECM overproduction, including collagen I and III, hyaluronic acid and laminin, decreasing the collagenase activity and collagen degradation [37,38]. Expression of α-SMA is a representative sign of activated stellate liver cells. An equally important role in the development of fibrosis is played by TGF-β1, which acts as the major profibrotic cytokine synthesized mainly by stellate liver cells [39]. The results of this study showed that the treatment with ORNs-d-M starting at 0 h after TAA administration is more effective in reducing the expression of α-SMA mRNA compared to the ORNs-d-M 12 h group. Also, our study showed a significant decrease in TGF-β1 and COL1A1 mRNAs that play a crucial role in the development of fibrosis. These results suggest that ORNs-d-M can have a protective effect on the formation of fibrosis after acute TAA-induced liver injury.

ORNs-d-M are complexes of total yeast RNA with a dominant fraction of 5–8 nucleotides and D-mannitol [40]. In our studies, we found that ORNs-d-M treatment has a protective effect in the model of TAA-induced acute toxic damage of the liver. We assume that the main mechanisms by which the complexes protect the liver from toxic damage are associated with their anti-inflammatory properties [10] and the ability to modulate some signaling pathways (including NF-κB signaling) [41] that are involved in the development of hepatotoxicity. We also believe that ORNs-d-M can directly affect the key molecules (including CYP2E1, CYP1A2, CYP2C6, CYP3A2) that are involved in TAA biotransformation. On the other hand, ORNs-d-M are capable of nonspecific binding to target molecules [42], so we assume that the complexes also can bind to a toxin, thereby preventing the development of hepatotoxicity. It is abundantly clear that the demonstration of the interdependence between our established effect and the possible ability of ORNs-d-M to affect both the signaling pathways involved in the development of hepatotoxicity and the TAA metabolism should be the subject of our further research.

## 4. Materials and Methods

### 4.1. Chemicals and Reagents

TAA was purchased from Sigma Aldrich, St. Louis, MO, USA.

ORNs-d-M complexes are compounds consisting of oligoribonucleotides (highly purified total yeast RNA) without protein and DNA impurities with a dominant fraction of 3–8 nucleotides and D-mannitol. The ratio of oligoribonucleotides to D-mannitol is 2.5:1 [40,43]. ORNs-d-M was purchased from the company “BioCell”, Kyiv, Ukraine.

All other chemicals were received from standard commercial suppliers (Sigma Chemical Co, Acros Organics, St. Louis, MO, USA; Thermo Fisher Scientific, Macherey Nagel, Berlin, Germany).

### 4.2. Animals and Experimental Design

All procedures that were performed in studies were in accordance with ethical standards (Federalwide Assurance No 00019663) and carried out within the guidelines of the European Convention for the Protection of Vertebrate Animals Used for Experimental and Other Scientific Purposes (Strasbourg, France, 1986) and with regard to the NIH Guide for the Care and Use of Laboratory Animals [44]. The experimental animals were female C57BL/6J mice of 2–2.5 months of age, with body mass of 20–22 g. Acute thioacetamide-induced hepatotoxicity was used to model toxic lesions of the liver in mice [45]. 200 mg/kg body weight dose of ORNs-d-M was chosen, as it was previously proven to be protective against oxidative damage caused by TAA in mice [46]. The animals (*n* = 6) were randomly divided into 4 experimental groups:

1. NaCl control group—the animals were administered normal saline (0.9% NaCl) by a single intraperitoneal injection.
2. TAA—the animals were given 500 mg/kg body weight of TAA by a single intraperitoneal injection.
3. ORNs-d-M 0 h—the animals were administered 500 mg/kg body weight of TAA by a single intraperitoneal injection, and then 200 mg/kg doses of ORNs-d-M (per os) immediately after TAA application (i.e., 0 h) and every next 12 h for 48 h (12, 24, 36, 48 h).
4. ORNs 0 h—the animals were administered 500 mg/kg body weight of TAA by a single intraperitoneal injection, and then 143 mg/kg (the quantity of ORNs in 200 mg/kg of the ORNs-d-M) doses of ORNs (per os) immediately after TAA application (i.e., 0 h) and every next 12 h for 48 h (12, 24, 36, 48 h).
5. d-M 0 h—the animals were administered 500 mg/kg body weight of TAA by a single intraperitoneal injection, and then 57 mg/kg (the quantity of D-M in 200 mg/kg of the ORNs-d-M) doses of D-M (per os) immediately after TAA application (i.e., 0 h) and every next 12 h for 48 h (12, 24, 36, 48 h).
6. ORNs-d-M 12 h—the animals were administered 500 mg/kg body weight of TAA by a single intraperitoneal injection, and then 200 mg/kg doses of ORNs-d-M (per os) after 12 h after TAA application (i.e., 12 h) and every next 12 h for 48 h (24, 36, 48 h).
7. ORNs 12 h—the animals were administered 500 mg/kg body weight of TAA by a single intraperitoneal injection, and then 143 mg/kg doses of ORNs (per os) after 12 h after TAA application (i.e., 12 h) and every next 12 h for 48 h (24, 36, 48 h).
8. D-M 12 h—the animals were administered 500 mg/kg body weight of TAA by a single intraperitoneal injection, and then 57 mg/kg doses of D-M (per os) after 12 h after TAA application (i.e., 12 h) and every next 12 h for 48 h (24, 36, 48 h).

At the end of the experimental period, the animals were anesthetized intraperitoneally with a ketamine–xylazine solution and euthanized after 48 h of TAA administration. 

### 4.3. Biochemical Determinations

GGT and ALT enzymatic activities were determined in mouse serum using a kit from Felicit Diagnostics (Felicit Diagnostics, Dnipro, Ukraine), according to the manufacturer’s instructions and expressed as U/l. MPO activity as an indicator of infiltration of parenchyma by neutrophils was determined by the method [47].

The evaluation of oxidative destruction of biomolecules was carried out on the basis of the determination of the levels of TBARS, protein carbonyl derivatives, protein thiol groups, and the level of reduced glutathione in the liver tissue. TBARS level was determined by a method [48] that is based on reaction between product of lipid peroxidation and thiobarbituric acid with production of a pink pigment with a 532-nm absorption maximum. TBARS content was expressed as nmol/mg of protein. The level of protein carbonylation was assayed with method [49]. This method is based on the reaction of protein carbonyls with 2,4-dinitrophenylhydrazine which leads to formation of a stable dinitrophenylhydrazone product. Carbonyl group levels was expressed as nmol/mg of protein. Protein thiol groups in liver were determined by reaction with Ellman’s reagent that produces yellow 2-nitro-5-thiobenzoate anion [50]. Hepatic reduced glutathione level was measured according to the method of Ellman [51].

An evaluation of the functional activity of the antioxidant system was analyzed for the enzymatic activity of glutathione peroxidase and glutathione-S-transferase. GPx activity in the liver was determined by method [52] and expressed in nmol/min/mg protein. GST activity was determined through the conjugation of GSH with 1-chloro-2,4-dinitrobenzene (CDNB) with a 340 nm absorption maximum [53].

Protein concentration was determined using the method of Lowry et al. [54].

### 4.4. RNA Preparation and Quantitative Real-Time PCR

The isolation of total RNA was performed using the NucleoMag RNA Kit (Macherey Nagel, Duren, Germany) and BeadRetriever system (Invitrogen, Carlsbad, CA, USA) according to the manufacturer’s protocol. The amount of isolated RNA and the presence of protein and carbohydrate impurities in it were determined by the absorbence of solutions at 260 nm and by the ratio A260/A280 using a MaestroNano Pro Micro-Volume MN-913 spectrophotometer (Maestrogen, Hsinchu, Taiwan). The integrity of total RNA was determined by the ratio of the intensity of the 28S/18S rRNA bands in the electrophoregram after electrophoresis using the Microchip electrophoresis system (MCE-202/MultiNA Shimadzu, Berlin, Germany). Synthesis of cDNA was performed in a final volume of 20 μL with a total RNA concentration of 2 μg using the Maxima H Minus First Strand cDNA Synthesis Kit (Thermo Scientific, Waltham, MA, USA). The reverse-transcription reaction was performed on Thermal Cycler CFX96 Real-Time system (Bio-Rad, Singapore) with the reaction parameters: 42 °C 60 min, 25 °C 5 min, 42 °C 60 min, 70 °C 5 min. Quantitative evaluation of IL-6, TNF-α, TGF-β1, COL1A1 and α-SMA gene transcription was performed by real-time polymerase chain reaction in a Thermal Cycler CFX96 Real-Time system (Bio-Rad, Singapore) using the BioRad CFX Manager software. The amplification was performed in the mode: 95 °C 10 min, 39 cycles: 95 °C 40 s, 60 °C 30 s, 72 °C 30 s, including the plate scanning. As a reference housekeeping gene used to normalize mRNA expression, we employed GAPDH. This gene gave excellent reproducibility, never varying in its Ct value by more than 0.5 units. To calculate the relative expression, the method 2^−ΔΔCt^ was used [55]. The primers sequenced were designed on GenBank database and were synthesized (Invitrogen, Carlsbad, CA, USA). Primers used for quantitative analysis of mRNA expression are shown in Table 2.

### 4.5. Statistical Analysis

All data are expressed as the mean ± SD. Student’s *t*-tests were used to compare different groups. The differences were considered significant if *p* ≤ 0.05.

## Figures and Tables

**Table 1:** Effects of ORNs-d-M, ORNs and d-M on the activities glutathione peroxidase (GPx) and glutathione S-transferase (GST) in TAA-induced liver damage mice.

Group	GPx Activity in Liver, nmol/min/mg of Protein	GST Activity in Liver, μnmol/min/mg of Protein
NaCl	276.66 ± 19.68 ^a^	1.21 ± 0.13 ^a^
TAA	146.62 ± 17.79 ^b^	0.68 ± 0.15 ^b^
TAA + ORNs-d-M 0 h	221.14 ± 21.70 ^c^	1.00 ± 0.16 ^c^
TAA + ORNs 0 h	201.31 ± 13.29 ^c^	0.92 ± 0.08 ^d^
TAA + d-M 0 h	135.19 ± 7.67 ^b^	0.58 ± 0.09 ^b^
TAA + ORNs-d-M 12 h	186.44 ± 4.40 ^d^	0.91 ± 0.07 ^d^
TAA + ORNs 12 h	158.18 ± 17.60 ^b^	0.64 ± 0.14 ^b^
TAA + d-M 12 h	139.69 ± 15.91 ^b^	0.54 ± 0.08 ^b^

Data are presented as the mean ± SD, *n* = 6 for each group. Values marked with different letters (a, b, c, d) are statistically different, *p* < 0.05 (Student’s *t*-test). Abbreviations: NaCl—control mice receiving a single injection of NaCl; TAA—mice receiving a single injection of TAA; ORNs-d-M 0 h—mice receiving a TAA and ORNs-d-M immediately after TAA application (i.e., 0 h) and every next 12 h for 48 h; ORNs 0 h—mice receiving a TAA and ORNs immediately after TAA application (i.e., 0 h) and every next 12 h for 48 h; d-M 0 h—mice receiving a TAA and then d-M immediately after TAA application (i.e., 0 h) and every next 12 h for 48 h; ORNs-d-M 12 h—mice receiving a TAA and ORNs-d-M after 12 h after TAA application (i.e., 12 h) and every next 12 h for 48 h; ORNs 12 h—mice receiving a TAA and ORNs after 12 h after TAA application (i.e., 12 h) and every next 12 h for 48 h; d-M 12 h—mice receiving a TAA and d-M after 12 h after TAA application (i.e., 12 h) and every next 12 h for 48 h.

**Table 2:** Primers used in this study.

Primer Name	Primer Sequence (5′→3′)
IL-6_for	5′-GTCACAGAAGGAGTGGC
IL-6_rev	5′-CTGACCACAGTGAGGAA
TNF-α_for	5′-CCTCCCTCTCATCAGTTCTA
TNF-α_rev	5′-CTTTGAGATCCATGCCG
TGF-β1_for	5′-GGCTACCATGCCAACTT
TGF-β1_rev	5′-ACCCACGTAGTAGACGA
COL1A1_for	5′-CCTCAGAAGAACTGGTACATCA
COLA1_rev	5′-GGCCTCGGTGGACATTA
α-SMA_for	5′-TCTGGCACCACTCTTTCTATAAC
α-SMA_rev	5′-TAGCCACATACATGGCGG
GAPDH_for	5′-TCAACAGCAACTCCCACTCTTCCA
GAPDH_rev	5′-ACCCTGTTGCTGTAGCCGTATTCA
